# Evidence that *Listeria innocua* modulates its membrane’s stored curvature elastic stress, but not fluidity, through the cell cycle

**DOI:** 10.1038/s41598-017-06855-z

**Published:** 2017-08-14

**Authors:** Samuel Furse, Martin Jakubec, Frode Rise, Huw E. Williams, Catherine E. D. Rees, Øyvind Halskau

**Affiliations:** 1 Department of Molecular Biology, University of Bergen, Thormøhlensgate 55, NO-5006 Bergen, Norway; 2 Centre for Biomolecular Sciences, University of Nottingham, University Park, NG7 2RD Nottingham, United Kingdom; 3 Department of Chemistry, University of Oslo, P. O. Box 1033, Blindern, NO-0315 Oslo, Norway; 4 School of Biosciences, University of Nottingham, Sutton Bonington Campus, LE12 5RD Nottinghamshire, United Kingdom

## Abstract

This paper reports that the abundances of endogenous cardiolipin and phosphatidylethanolamine halve during elongation of the Gram-positive bacterium *Listeria innocua*. The lyotropic phase behaviour of model lipid systems that describe these modulations in lipid composition indicate that the average stored curvature elastic stress of the membrane is reduced on elongation of the cell, while the fluidity appears to be maintained. These findings suggest that phospholipid metabolism is linked to the cell cycle and that changes in membrane composition can facilitate passage to the succeding stage of the cell cycle. This therefore suggests a means by which bacteria can manage the physical properties of their membranes through the cell cycle.

## Introduction

The bacterial cell cycle is an example of binary fission that is typically both rapid and reliable. It is characterised by periods known as B, C and D^1–4^. The B period represents the phase between division and the initiation of replication, whereas the C period (sometimes called the replication phase) represents the phase in which the nucleolus is replicated and in which the bacterium reaches its mature size. The D period is that between the replication of the DNA and fission of the cell envelope. The B, C and D periods can therefore considered analogous to the eukaryotic G_1_, S and G_2_ phases, respectively. However, despite considerable research, little is understood about how progress from the B to D periods is controlled.

In Eukaryotes it is known that the cell cycle is controlled through the interaction of cyclin and cyclin-dependent kinases^5–7^. However, to date there is no evidence for the presence of homologues of cyclin in bacteria. Furthermore, studies of Gram-negative species have been unable to produce evidence for regulation of cell division either through changes in gene expression^8^ or changes in the concentration of the proteins that comprise the divisome^9, 10^. Existing models for the control of bacterial cell division allow for several possible mechanisms. One is based on regulation of increase in cell size (the size adder theory^11–13^). Another is that cell size is regulated in advance of division, possibly through dilution of a ‘timekeeper’ protein, though the mechanism underlying this model is not known^13, 14^ and it is not clear whether such proteins would control, or are rate-limiting, with respect to the cell cycle. There are also hints that cell structure is a determinant in controlling the binary fission in bacteria^15^. In principle, control of progress through the cell cycle through changes in cell structure could be based on both structural proteins and lipids, but knowledge of the role of lipids in this is lacking.

Recent work on lipids and their metabolism in the Gram-positive bacterium *Bacillus subtilis* has revealed a link between the synthesis of phospholipids and the divisome. This evidence suggests that *de novo* synthesised lipids are produced at the septum during fission^16^. Furthermore, there is evidence for an overall change in the lipid profile of the Gram-negative *Escherichia coli* through its cell cycle^15^. The latter work showed that the abundance of curvature-forming cardiolipin (CL) falls whilst that of the bilayer-forming phosphatidylglycerol (PG) increased on elongation of the cell at the C/D boundary. Evidence from biophysical studies conducted over the last half-century shows that modulation of lipid composition can have a considerable impact on the lyotropic phase behaviour of lipid systems^17, 18^. For example, an increase in the fraction size of phosphatidylethanolamine (PE) increases the propensity of a lipid system to assemble into curved phases rather than bilayer-like ones^19, 20^. This is known as increasing stored curvature elastic stress, SCES^21^.

Determining the relationship between lipid composition and phase behaviour complements the interpretation of lipid profiling studies. Data from studies of the phase behaviour of lipids suggest that the increase in abundance of a bilayer-forming lipid and the reduction in abundance of a non-bilayer forming lipid that occurs in *E. coli* on elongation, reduces SCES^22–25^. This is consistent with the changes in morphology of the cell during elongation because average curvature is lower in elongated cells and the evidence that the location of the Z-ring may be determined by cell membrane geometry^26–28^. This led us to the hypothesis that the production and shape of the cell envelope is involved in controlling the cell cycle of bacteria. If correct, this hypothesis implies that the topology of the plasma membrane and thus the physical properties of its lipids play a significant role in progress through the cell cycle in bacteria.

In order to investigate whether modulation of lipid composition through the cell cycle is a general feature of bacteria we measured global changes in the lipid profile of a bacterium that is evolutionarily distinct from *E. coli*. *Listeria innocua* NCTC 11288 was chosen because, unlike the commonly studied model Gram-positive *B. subtilis*, it does not sporulate under stress. *L. innocua* also has clinical relevance^29^, due to its biological similarity to the pathogen *L. monocytogenes* ^30, 31^.

Cultures of *L. innocua* in the exponential phase were stopped after elongation but before cell division (around the C/D period boundary) with the bacteriostatic antibiotic rifampicin (RIF) using an adapted^15^ version of an established method^32–34^. The lipid fractions (LF) were isolated from freeze-dried, pelleted cultures whose cell walls had been thoroughly digested. The LFs were profiled using a combination of solution phase ^31^P NMR and HR-MS/MS^35^. The relationship between changes in lipid composition and membrane morphology observed in *L. innocua* was investigated using broad line ^31^P NMR to characterise the phase behaviour of model systems that corresponded to the lipid compositions observed.

## Results

### Culture growth and synchronisation

**Figure 1:**
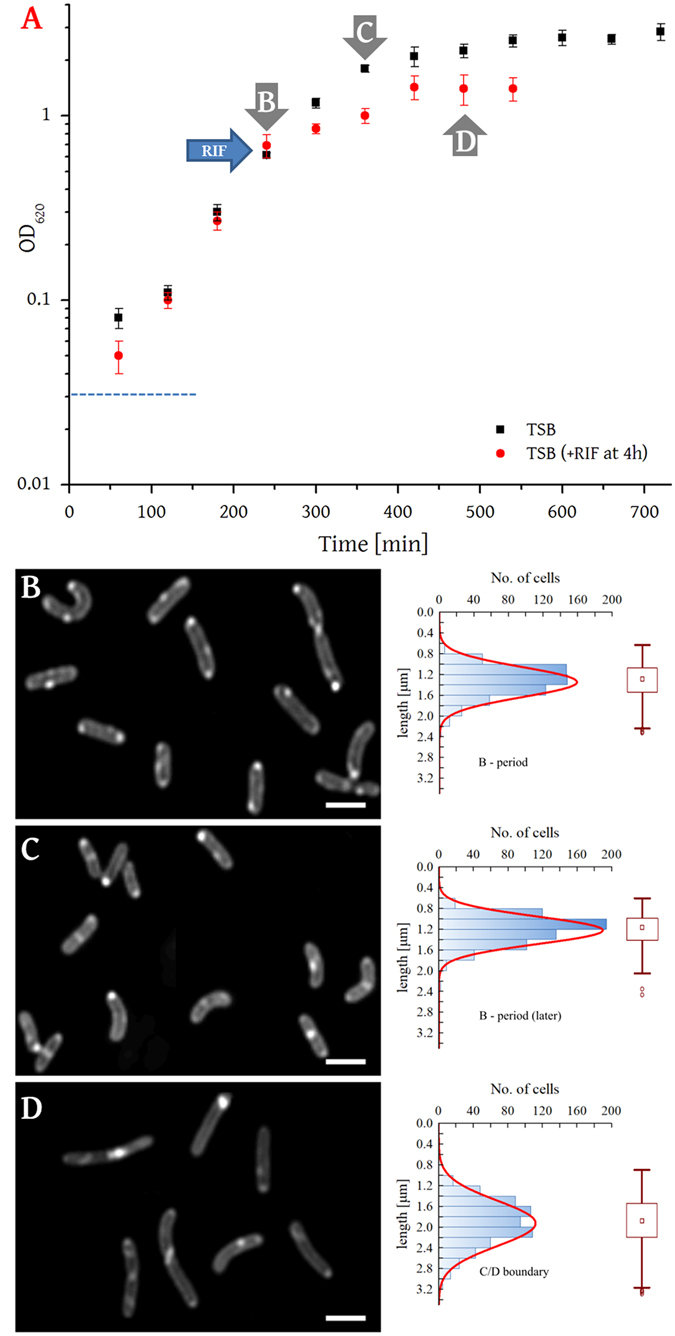
Identification of growth phases and image analysis of cultures. Panel A, Growth curve for L. innocua NCTC 11288 grown in TSB at 37 °C (*n* = 3 independent experimental measurements per point). The lag phase lasted for approximately 150 min (------); exponential phase (untreated cultures) began at about 150 min and by 400 min the cells were in the stationary phase. In cultures treated with RIF, stationary phase was induced immediately although some growth was still detected for ~120 min. Samples in panels B and C (untreated controls) were collected after 240 and 360 min and sample in panel C was collected 240 min after RIF administration (at 480 min). Panels B–D show light and fluorescence micrographs of *L. innocua*; for fluorescent imaging samples were stained with Nile red (1000 × magnification; scale bar = 1 µm). A graph of the distribution of cell lengths is shown for each sample with Gaussian distribution (red line). Data represents 180–200 cells from at least three images from four independent cultivations. The Box plots show medians, 50% of data in the range (box), non-outliers range (whiskers) and outliers (circles and stars). Sample B is representative of cells in exponential phase growth (240 min after inoculation) with the majority of cells appearing to be in the B period (>80% synchrony; mean = 1.34 µm, ± 0.29 µm). Sample C is representative of cells at the end of the exponential phase (360 min after inoculation) and represents the B period of older cultures (>80% synchrony; mean = 1.22 µm, ±0.26 µm). Sample D is representative of cells arrested at the C/D boundary by RIF (80-90% synchrony; mean = 1.92 µm, ±0.44 µm). Only 6.7% of cells in the C/D boundary samples are as short or shorter than those seen to predominate in the untreated samples (t-test, p =  < 0.01).

Cultures of *L. innocua* NCTC 11288 were grown in tryptic soya broth at 37 °C and the growth phase synchronised using RIF^15^. A growth curve was used to identify the appropriate point to administer the arrest drug (determined empirically to be 240 min (Fig. 1).

The experimental steps for preparation of samples and collection of profiling data are shown in Fig. S1. Based on their size distribution the synchrony of the untreated exponentially growing cultures was determined to be >80% (and the cell size analysis suggested that these were predominantly in the B period) (Fig. 1). The cell size was measured using cells stained with Nile Red observed using fluorescence microscopy. These data were used to establish the efficacy of RIF in arresting and holding cells at the C/D boundary and cells from such cultures were found to be significantly longer (mean = 1.92 µm, ± 0.44 µm) than those in the untreated samples (mean = 1.34 µm, ± 0.29), with only 6.7% being as short or shorter than those seen to predominate in the untreated samples (*t*-test, *p* *=* *<* 0.01). Based on these results the untreated samples were taken to be representative of cells in the B period, and the RIF-treated samples representative of cells at the C/D boundary.

We took several steps in order to avoid chemical and enzymatic degradation of lipids. After centrifugation cells were resuspended in PBS to suppress PI-PLC activity^36^, and a mixture of lipase inhibitors was added to suppress the activity of these enzymes *ex vivo* ^15, 35^. A mixture of mutanolysin^37^, lysozyme and RNAase was added to digest the cell wall and thus maximise access to the lipid fraction^38^.

Possible contamination of the cultures was checked for by inspecting the ^1^H spectrum of the FA fraction but none was found (that of *Listeria ssp.* ^39–42^ is distinct from other commonplace bacteria, such as *E. coli* ^43–46^. See *Preparation of fatty acid isolates* in *Experimental Methods*, and Fig. S2).

### Isolation of the lipid fraction

The LFs were extracted using a two-step method that was designed to address the apparent short-comings of traditional methods^47^ (see *Experimental* for details). Triethylammonium ions were used to provide a counter-ion that is soluble in organic solvents and thus favours migration of phospholipid species into the organic phase. Dichloromethane was used in place of chloroform in order to minimise chemical degradation of lipid species^47^. Lastly, in order to maximise the extraction efficacy, the freeze-dried free-flowing powdered cellular matter was washed in a mixture of organic solvents (dichloromethane/methanol/triethylammonium chloride, 3:1:0.0005, *v/v/w*) before being dispersed in water and methanol, and extracted with dichloromethane. In order to test the effectiveness of this procedure, the aqueous fraction and precipitate from the isolation of the LF were freeze-dried and treated with strong acid (5% H_2_SO_4_) in order to hydrolyse any FA ester bonds and thus release any remaining FAs. The amount of FA found is treated as proportional to the mass of the lipid not collected during the isolation. The data from these measurements (^1^H NMR, see *Experimental* and Fig. S3) suggest that the procedure used in this study extracted >99% of the LF. This compares favourably with around 85% for the original Bligh & Dyer method^48^.

### Profiling of the lipid fraction

**Figure 2:**
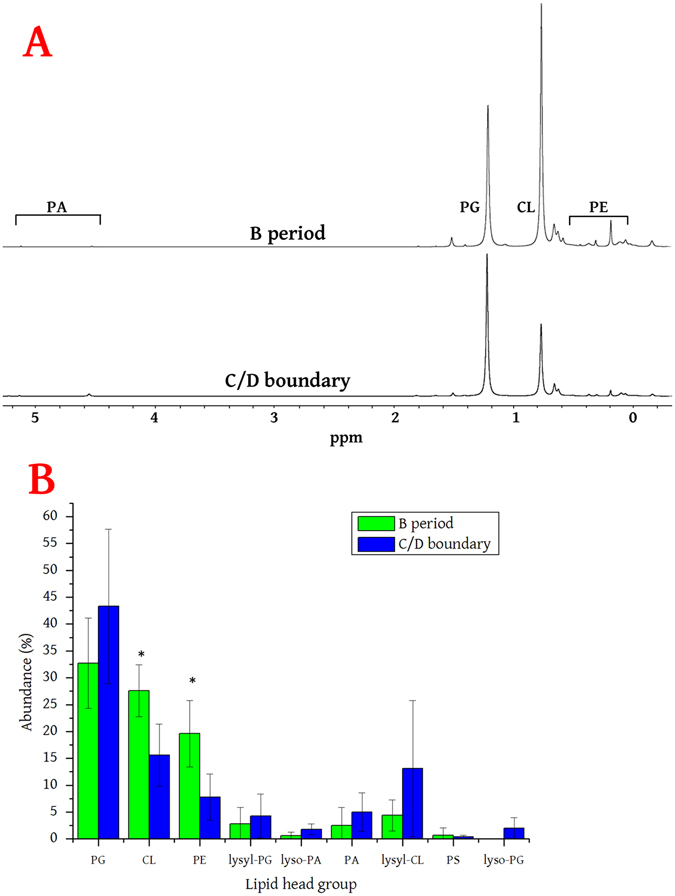
Profiling of the phospholipids in *L. innocua* NCTC 11288 at different stages of the cell cycle. Panel A shows representative ^31^P NMR spectra of the lipid fraction collected from B period cultures and cultures at the boundary of the C and D periods. The relative area of the integrations of the appropriate resonance(s) were taken as a fraction of the total integrations for each spectrum and used to generate the fraction size given for each sample. The shift of phosphate *mono*-ester-containing lipids such as PA, is pH dependent. PE exhibits several resonances due to concentration and pH-dependent solvent interactions^15, 78^. Panel B shows the abundance of lipids in the B period and at the C/D period boundary from cells collected in the exponential phase. Asterisks mark the head groups (CL, PE) for which the difference in abundance is considerable (when comparing standard deviations, *n* = 5 independent samples profiled using quantitative ^31^P NMR). CL, cardiolipin; *lyso*-PA, lyso-phosphatidic acid; *lyso*-PG, *lyso*-phosphatidylglycerol; lysyl-CL, lysyl-cardiolipin; lysyl-PG, lysyl-phosphatidylglycerol; PA, phosphatidic acid, PE, phosphatidylethanolamine; PG, phosphatidylglycerol; PS, phosphatidylserine.

The LFs were profiled using a combination of solution phase ^31^P NMR and HRMS/MS^35^. ^31^P NMR was used for quantification because it allows non-destructive and unambiguous high-resolution spectroscopy of the organically-soluble phosphorus-containing molecular species present. Lysylated lipids have not been profiled using ^31^P NMR before and so the shifts of lysyl-dioleoyl phosphatidylglycerol (lysyl-DOPG) were determined (Fig. S4). Representative spectra of the lipid profiling and the relative abundance of lipids in the B period and at the C/D boundary are shown in Fig. 2A. A combination of high resolution and tandem mass spectrometry were used to identify both the head groups present and the range of lipid isoforms present (Table S1 shows the lipid abundance and isoform profiles. Representative lipid fragmentation is shown in Fig. S5).

These data show that the abundance of both cardiolipin (CL) and phosphatidylethanolamine (PE) fall significantly between the B period and at the C/D period boundary. The fraction size of CL falls by around 40% whereas that of PE falls by about 60%. The absolute value for the abundance of PG varies between cultures, but does not appear to be statistically significant. This is perhaps surprising in view of the significant decreases in both CL and PE, however it is not clear why this should be. It seems unlikely that this is due to lysylation as the abundance of lysyl-PG is also not statistically significant, despite notable shifts in the absolute values. The abundance of major structural lipids CL and PE does not change substantially between cultures at the beginning and end of the exponential phase, while PG changes to some extent (Table S1). Unlike *E. coli*, the age of cultures of *Listeria* does not appear to correlate with changes in its lipid profile.

Profiling of the fatty acid residues (FARs) of the total lipid fraction did not indicate a difference in the FA profile between periods, suggesting that FA production is not linked to the cell cycle in this organism (Fig. S6). These data therefore indicate that although the lipid profile changes through the cell cycle of *L. innocua* it does not change with the age of rapidly-dividing cells. Furthermore, cell-cycle dependent changes are restricted to the head groups of the major structural species. Taken together, these data suggest that synthesis of the principal head groups in particular is linked to the expansion of the membrane during the B and C periods.

These data (Fig. 2B, Table S1) are consistent with the published evidence that the bulk of the *Listeria* LF comprises PE^49^ and lysyl-derivatives of PG and CL^50^, but is at odds with reports in which PE appears not to have been found^39, 50^. Conversely, phosphatidylinositol (PI) was not detected in any sample in this study despite the presence of several PLC inhibitors, contrasting with reports of the presence of PI in a closely-related strain of *Listeria* ^42, 51^. We note that the lipid profile of biological samples can be influenced by myriad factors, including the medium used, how cultures are incubated and handled, how the LF is isolated, handled and profiled, and even the activity of endogenous lipases *ex vivo* ^47^.

### Lyotropic phase behaviour of model membrane systems

The evidence for modulations in the abundance of CL and PE during cell elongation raises the question of whether membrane properties change with these changes in composition. Studies of the phase behaviour of PG, PE and CL over the last half-century have shown that they have distinct properties. PE’s spontaneous curvature is negative^19, 52–54^, as is CL’s but only in the presence of a high concentration of divalent cations^23, 55, 56^. PG’s phase behaviour is dominated by lamellar phases, suggesting that its spontaneous curvature is negligible^24, 25, 57–59^. Modulation of the abundance of PE and CL (with a low concentration of divalent cations) is therefore a change in the abundance of two major structural lipids with contrasting spontaneous curvature. That these changes in abundance occur through the cell cycle therefore suggests that the stored curvature elastic stress of the membrane also changes through the cell cycle. We investigated this by modelling the behaviour of two-lipid mixtures using broad line ^31^P NMR.

Model systems comprised the di-oleoyl isoforms of PG and PE (DOPG and DOPE), and tetra-oleoyl isoform of CL (TOCL). Temperature scans of the individual lipids revealed the established behaviour, *i.e*. systems comprising either TOCL or DOPG showed fluid lamellar (L_α_) phase over the temperature range studied (Fig. S7A,B), with DOPE undergoing a transition from L_α_ to inverse hexagonal (H_II_) only above 273 K (Fig. S7C).

**Figure 3:**
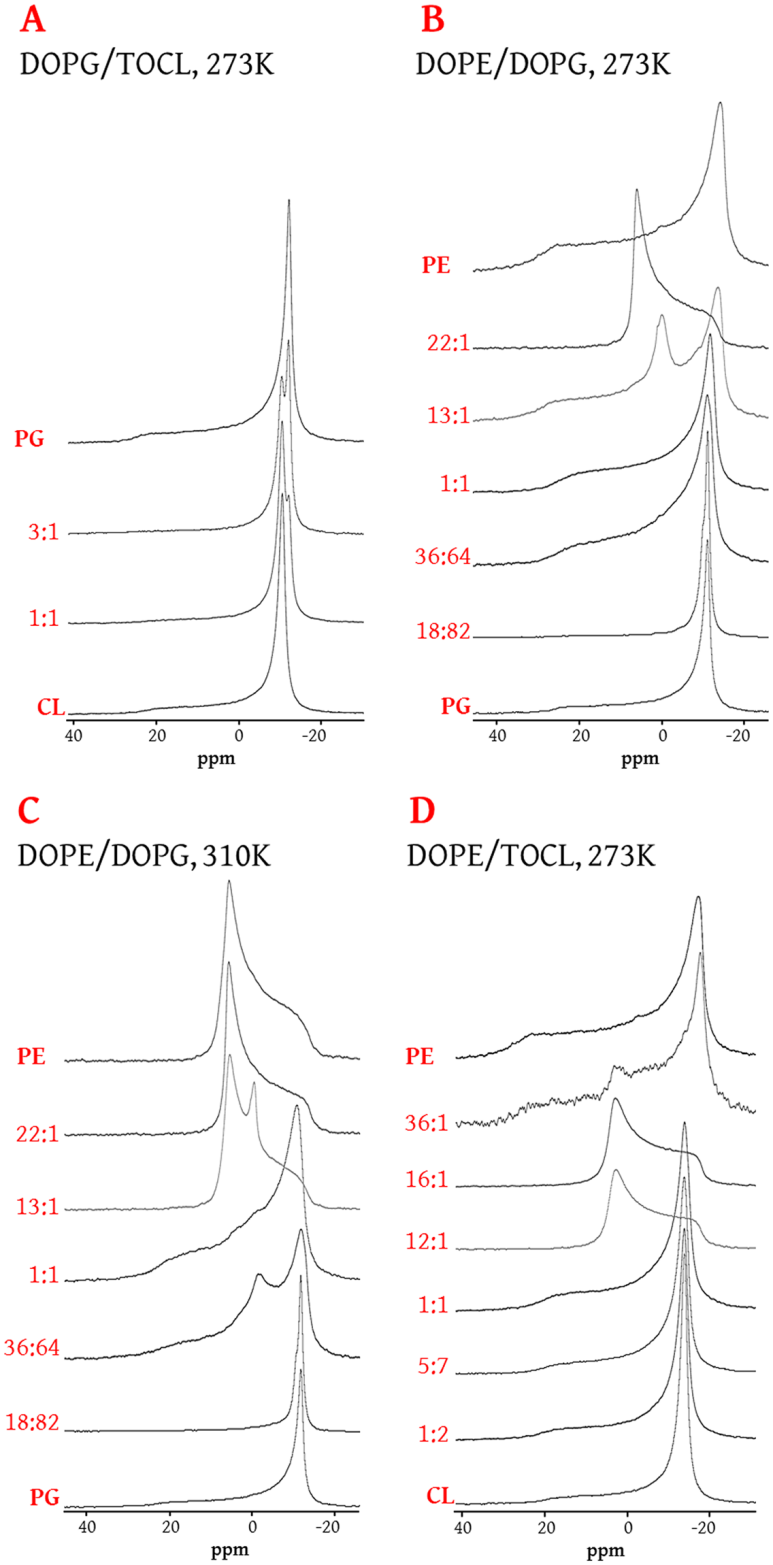
Stacked spectra showing the phase behaviour of two-lipid systems as a function of lipid head group composition. (**A**), DOPG/TOCL mixtures at 273 K; (**B**), DOPE/DOPG at 273 K; (**C**), DOPE/DOPG at 310 K; (**D**), DOPE/TOCL scan at 273 K. The 36:64 and 18:82 mixtures of DOPE:DOPG represent the ratio of these two lipids in the B period and at the C/D boundary, respectively. DOPG, dioleoylphosphatidylglycerol; TOCL, tetraoleoylcardiolipin; DOPE, phosphatidylethanolamine. Full temperature scans of each lipid mixture can be found in Figs S8–10.

Mixtures of DOPG with TOCL show a strong preference for L_α_, even at relatively low temperatures (273 K, Fig. 3A; Fig. S8A–C), suggesting that both lipids confer fluidity on systems in which they are present. The L_α_ phase also dominates across most of the concentration range in mixtures of DOPG and DOPE at 273 K (Fig. 3B), but higher temperatures favour curved phases (*e.g*. 310 K, Fig. 3C; Fig. S9). These data indicate that PE confers stored curvature elastic stress on PG systems. In particular, a reduction in PE from 36% to 18%, similar to that on elongation of *L. innocua*, reduces the ability of that system to form curved phases, suggesting a significant reduction in SCES. The L_α_ dominates in mixtures of DOPE with TOCL where the concentration of DOPE is below 50% (273 K, Fig. 3D). This also indicates the preference for lamellar phase(s) by TOCL at low concentrations of divalent cations (Fig. S10A), and curved phases by PE (Fig. S10C). Thus, although the fraction of CL shrinks on elongation of *L. innocua*, the increase in that of the DOPG ensures that the reduction in the abundance of PE reduces SCES.

However, it appears that doping DOPE with rather small quantities of DOPG (22-13:1, 4.3-7.1%) or TOCL (~36-12:1, 2.7-7.7%) allows the formation of curved phases at 273 K, particularly the H_II_ phase (Figs 3B and S9, and Figs 3D and S10). Importantly, the phase transition from fluid lamellar to the H_II_ phase occurs in DOPE around 10 K higher than this, at ∼ 281K^60^. This suggests that the presence of small quantities of these lipids can relieve the packing stress of the H_II_ phase.

Data from a mixture of lysyl-DOPG with TOCL suggest that lysyl-DOPG has similar fluid properties to DOPG (Fig. S8C and D) but does not reduce the packing stress of DOPE systems as DOPG does (Fig. S9E and F). Notably, the behaviour of DOPE:lysyl-DOPG (22:1, Fig. S9F) is very similar to that of pure DOPE (Fig. S7C), and both are distinct from DOPE:DOPG (22:1, Fig. S9E). In the latter, the less abundant lipid appears to reduce the packing stress of the inverse hexagonal phase. This suggests that the role of lysyl-DOPG in the membrane differs from that of PG and may be related more to modulations in SCES and/or lipid packing than to fluidity.

## Discussion

The evidence from this study indicates that the modulations in lipid composition in *L. innocua* that occur between the B period and C/D boundary are statistically significant. The behaviour of model systems suggests that the modulations in lipid composition on elongation of *L. innocua* represent a reduction in the SCES of the membrane (observed as a reduction in size of PE fraction, Fig. 3B) but without a reduction in its fluidity (with an increase in PG and despite a decrease in CL; Fig. 3A and D). This suggests that the physical properties of the membrane are linked to the cell cycle.

The behaviour of model lipid systems observed in this study supports the conclusion that changes in lipid composition favour changes in morphology of the membrane on a cellular scale. Comparison of the lipid profile and distribution between different rod-shaped bacteria (such as *E. coli* and *B. subtilis*) and between rod-shaped and spherical bacteria (such as *Staphylococcus aureus*) are therefore of interest in understanding the cellular structure of prokaryotes. However, comprehensive cell-cycle-based lipid profiling of prokaryotes is lacking at present. What is known, is that *E. coli* and *B. subtilis* have very different distributions of lipids in their membranes, which are linked to differences in proteins associated with cell division, and therefore cell shape is not expected to be the only factor that determines this change^61^.

We note that the modulations in lipid composition of the Gram-positive *L. innocua* are analogous to those reported for the Gram-negative bacterium, *E. coli*. In the latter organism, the abundance of the bilayer-forming PG increases during its elongation (from ~1:22 to ~1:16 against PE), with a reduction in the abundance of CL (from ~1:16 to ~1:36 against PE)^15^. Physical data from the present study suggest that there is also a reduction in the tendency to form the hexagonal phase (lower SCES) in *E. coli*, without a reduction in fluidity on progression from the B period to the C/D boundary. This indicates that *E. coli* has adopted the same pattern with respect to modulation of lyotropic phase behaviour between the B period and C/D boundary as seen in *L. innocua*.

This remarkable similarity between two bacteria that are quite separate in evolutionary terms raises the question of whether such modulations are important for the structure of rod-like cells in general. However, it is not clear from these data whether this trend is limited to cells with a cell wall; further work is required to establish what effects modulations in lipid composition have in other cell types.

It is appealing to speculate what further effects the modulations in lipid composition may have on membrane properties. The folding and function of proteins of both bacterial and eukaryotic origin have been shown to be sensitive to lipid membrane composition^62–64^, and to SCES and packing defects^65, 66^. This coupling between the membrane’s physical state and the proteins’ folding can also manifest itself in problematic rather than functional ways. For instance, there is evidence that charged head groups such as those of PG and phosphatidylserine enhance the membrane binding and mis-folding behaviour of α-Synuclein^67^, and that packing defects are involved in promoting mis-folding of a protein linked to Parkinson’s disease^68, 69^. Particular constellations of ganglioside and cholesterol have also been proposed to promote both membrane binding and mis-folding in α-Synuclein and β-amyloid peptide^70^.

It is clear that further work is required to fully characterise the relationship between lipid composition and distribution in bacteria^71^ and in eukaryotic^72^ cells. Data such as that acquired in this study indicate the extents to which the lipid composition and phase behaviour vary with the cell cycle. These alterations may in turn have an impact on the structural and functional properties of the membrane they comprise.

## Experimental Methods

### Reagents and chemicals

Growth medium, solvents and fine chemicals were purchased from *SigmaAldrich* (Gillingham, Dorset, UK) and used without purification. PhosSTOP tablets were purchased from Roche (Welwyn, Hertfordshire, UK) and stored at 4 °C. Purified lipids were purchased from *Avanti Polar lipids Inc*. (Alabaster, Alamaba, US) and used without further purification.

### Cultivation of *L. innocua*

The growth kinetics of this bacterium in tryptic soya broth (TSB) was characterised over 24 h (Fig. 1). The preparation of one set (*n* = 1) of cultures is described. Each sample comprised three cultures in order to ensure that enough material was produced and to account for differences between populations. Thus for each biological replicate, 3 × 3 × 1 L cultures were grown in unmodified TSB that had been autoclaved. Mini-cultures (10 × 10 mL) were incubated (16 h, 37 °C, orbital incubator, 250 r.p.m.,) before inoculating 9 × 1 L to give cultures of OD_620_ ~0.03. After incubation (37 °C, 240 min, orbital, 250 r.p.m., average OD_620_ 0.4), three cultures were mixed together to make the 4 h control time point and cells harvested (fixed-angle rotor, 6k × *g*, 10 min, 4 °C). Three cultures were treated with RIF (50 mg/L final concentration, added as methanolic solution of 50 mg/mL). After 360 min h, the three remaining control cultures were mixed and harvested together (making the 6 h control time point which is equivalent to the point at which the cultures treated with RIF are arrested), and after 480 min the remaining three cultures treated with RIF were harvested together.

### Fluorescence and light microscopy

Cells were photographed after harvesting and before the administration of lipase inhibitors. Cells were stained with Nile Red as previously described^73–75^. Briefly, cells were collected by centrifugation (fixed-angle rotor, 6 k × *g*, 10 min, 4 °C), washed once with PBS and resuspended in PBS at OD_620_ ~2.0. Nile Red (2 µg/mL, sample volume 1 mL, dye stock solution in DMSO) was added and the sample (1 mL) allowed to stand at room temperature (20 min). Cells (60–80 per picture, 180–200 per sample) were measured from at least three images for each culture/time point each from four independent cultivations (*i.e*. (~70 × 3) × 3 × 4). The mean and standard deviation were calculated for each and an independent student *t*-test was used to determine statistical significance (*p* = < 0.01). The null hypothesis was that the lengths of the bacteria are the same for each sample. Images were taken using a Leica DMI6000 B microscope equipped with a Leica DFC350 FX camera and processed with (the 3D deconvolution function in) Leica Application Suite, Advanced fluorescence 1.7.0 software with AF6000 configuration and open source ImageJ software.

### Isolation of lipid fraction

Fresh cell pellets were resuspended immediately (PBS, 5 mL) and treated with PhosSTOP (1 tab/10 mL final concentration, 10 × stock dissolved in PBS), 2-butoxyphenylboronic acid (BPBA, 2 mg/mL final concentration, ethanolic stock solution 100 mg/mL) and a stored mixture of mutanolysin (50 µg), lysozyme (10 mg), RNAase (200 µg) (volume of aliquots 1 mL, glycerol/PBS 1:1, stored at 193 K). The total volume of the resulting solutions was 10 mL per sample and they were agitated gently in air tight falcon tubes (50 mL, gel staining table, 16–20 h) before being frozen (193 K) and freeze-dried. The freeze-dried material was stored at 253 K until isolation of the lipid fraction.

The free-flowing powdered cellular matter was mixed with dichloromethane/methanol (20 mL, 3:1, 0.5 mg/mL triethylammonium chloride, TEAC) and sonicated (sonication bath, 5 min) before being centrifuged (5 min, 5.25 k × *g*). The organic solution was decanted and retained. The pellet was mixed with more of the same dichloromethane/methanol mixture (20 mL, 3:1, 0.5 mg/mL TEAC), agitated, centrifuged and the solvent decanted. The pellet was then resuspended in a mixture of dichloromethane and water (20 mL, 1:1, separating funnel) and diluted with sufficient methanol to make a stable uniphasic solution (~40 mL), and agitated briefly. The mixture was then made biphasic by addition of dichloromethane (20 mL). The dichloromethane solution was removed and the aqueous solution washed (dichloromethane, 20 mL). TEAC was added to the remaining aqueous solution (final concentration of 2 mM, 2 M stock) and the aqueous solution washed with dichloromethane (2 × 20 mL). The combined organic solutions (~130 mL) were filtered through filter paper and concentrated *in vacuo* before storage under nitrogen at 253 K.

### Isolation of residual lipidic material

The aqueous phase and precipitate produced during the lipid isolation (above) were freeze-dried together and then treated with acid (H_2_SO_4_, 5%, 20 mL, gentle agitation, 24 h, r.t.p.). The reaction mixture was quenched (NaHCO_3_) and freeze-dried. The free-flowing powder was washed twice with chloroform. The organic phases were combined and concentrated *in vacuo*. The remaining material (~8 mg/120 mg LF isolated) was dissolved in deuterated chloroform (650 µL) before commencing ^1^H NMR. The integrals of each spectrum were calibrated according to the α-CH_2_ signal (2.3–2.4 ppm) present (Fig. S4).

### Solution phase ^31^P NMR spectroscopy

Lipid films (10-15 mg) from cultures of *L. Innocua* were dissolved in the CUBO solvent system^15, 35, 76^ (500 µL/sample). Data acquisition was similar to published work^15, 35^ but using a Bruker 400 MHz Avance III HD spectrometer equipped with a 5 mm BBO S1 (smart) probe operating at 298 K for all data except that shown in Fig. S5. ^31^P spectra were acquired at 161.98 MHz using inverse gated proton decoupling, with 2048 scans per sample and a spectral width of 19.99 ppm. An overall recovery delay of 6.5 s was used which gave full relaxation. Data were processed using line broadening of 1.00 Hz prior to zero filling to 19428 points, Fourier transform and automatic baseline correction. The data for Fig. S5 were acquired on a Bruker Avance III 800 MHz spectrometer, equipped with a QCI cryoprobe probe. Acquisition used inverse gated proton decoupling. Spectra were averaged over 1312 transients with 3882 complex points with a spectral width of 14.98 ppm. An overall recovery delay of 8.4 s was used. Data were processed using an exponential line broadening window function of 1.5 Hz prior to zero filling to 32768 points, Fourier transform and automatic baseline correction. All spectra were processed and analysed using (the dcon function in) TopSpin 3.2.

### Preparation of fatty acid isolates

Representative samples used for head group profiling were dried *in vacuo* (high-vac pump) before being diluted (H_2_SO_4_ 97% in methanol, 1:20 *v/v* final concentration, 5 mL) and heated under intermittent agitation (4–6 h, 50 °C). The reaction mixture was quenched (NaHCO_3_, 1 g) before being diluted (H_2_O, 30 mL; diethyl ether, 20 mL). The mixture was shaken vigorously in a separating funnel before the organic fraction was collected. The aqueous solution was washed (diethyl ether, 20 mL). The organic solutions were combined and dried *in vacuo*. The resulting oil (5–10 mg) was dissolved (CDCl_3_, 500–600 µL) and subjected to ^1^H NMR spectroscopy. δ_H_ (500 MHz, 16 scans, CDCl_3_, calibrated to 7.24 ppm) 3.6 (singlet, RCH_2_CH_2_COOC*H*_*3*_), 2.3 (triplet, *J* = 7.6 Hz, RCH_2_C*H*_2_COOCH_3_), 1.6-1.5 (m, RC*H*_*2*_CH_2_COOCH_3_), 1.4-1.0 (m, methylenes), 0.9-0.8 (m, methyl groups) ppm.

### Mass spectrometry

Samples were prepared and measurements taken according to published methods^15, 35^. Accurate mass LC-MS and MS/MS was performed on an OrbiTrap Dionex 3000, (Waltham, MA, USA). Dry lipid mixtures were dissolved in dichloromethane/isopropanol 1:1 and an inject volume of 10 µL was used. Analytes were separated chromatographically on a UPLC C18 column (1.7 µm particle size, Waters) at 40 °C at a rate of 0.4 mL/min. Mobile phase A consisted of 0.1% formic acid in water at *p*H 6.0, and mobile phase B was 56% acetonitrile, 40% isopropanol and 5% water with 0.1% formic acid. Ions were monitored in positive mode (*m/z* range 300-2000, resolution 140,000). Selected peaks were fragmented (normalised collision energy set to 36) and MS2 collected with a resolution of 17,500.

Analyses consisted of searches for lipid isoforms in MS1 spectra, followed by fragmentation (MS2) for appropriate *m/z* values. Lipid isoform searches were restricted by head group (PG, PE, PA, PS and CL), FAR (chain length from 10 to 20 C, up to three double bonds) and adduct (H^+^, Na^+^, K^+^, NH_4_^+^). The search was augmented to include lysyl and *lyso*- isoforms. Matches were compared (see Fig. S6 for example). The software-generated scores were checked manually. Analyses were performed using Thermo Xcalibur 3.0.63 and recently-developed software by Kochen *et al*.^77^. Original code for non-standard head groups was written by us in Matlab R2015b.

### Broad line ^31^P NMR sample preparation

Lipid mixtures were made in solution (dichloromethane, 10–15 mg/mL) and dried to a lipid film *in vacuo*. Dried lipid films were dispersed in aqueous buffer (NaCl 100 mM, tris 50 mM, CaCl_2_ 2.5 mM, MgCl_2_ 2.5 mM, *p*H 7.4; 30:1 *v/w*) with sonication and agitation. Deuterium oxide (10% *v/v* against buffer) was added and the mixture agitated and then freeze-thawed 8–10 times. Samples were then stored at 193 K, transported at ~295 K and were stored at 253 K before running.

### Broad line ^31^P NMR spectroscopy

A Bruker Avance III HD 400 MHz DRX spectrometer equipped with a Bruker BBO S1 (smart) probe was used for proton-decoupled broad line ^31^P NMR experiments. Experiments were performed at 161.98 MHz with an inverse-gated pulse sequence with proton decoupling during acquisition, spectral width of 200.44 ppm, acquisition time of 0.299 s, pre-scan delay (DE) time of 20 µs, receiver gain of 203 with 19428 data points and 2048 scans per spectrum. The duration of each scan, acquisition time and relaxation delay, was 2.332 s. Spectra were processed using TopSpin 3.2 and 3.5 with line broadening of 50 Hz, phase correction and automatic baseline correction (abs).

### Broad line ^31^P NMR temperature scans

Samples were brought to and held at the desired temperature for 15 min before acquisition. Temperature scans consisted of acquisitions at the following temperatures: 298, 273, 293, 298, 310, 318, 273, 310 K. The latter two temperature points were used to assess the reliability of the equilibration time used (15 min). Acquisitions at 338 K were carried out separately with equilibration and acquisition at 310 K immediately beforehand. Stackplots of spectra were prepared manually from individual traces processed as above.

## Electronic supplementary material

Supplementary information
