# Supplementary material for: Evidence that Listeria innocua modulates its membrane’s stored curvature elastic stress, but not fluidity, through the cell cycle
Source: Sci Rep. 2017 Aug 14;7:8012. doi: 10.1038/s41598-017-06855-z (PMC5556093; doi:10.1038/s41598-017-06855-z)
Supplement: Supplementary file 1 — Supplementary information [file 41598_2017_6855_MOESM1_ESM.pdf]

Supplementary Information

For: Evidence that *Listeria innocua* modulates its membrane's stored curvature elastic stress, but not fluidity, through the cell cycle

Samuel Furse[§], Martin Jakubec[§], Frode Rise‡, Huw E. Williams[†], Catherine E. D. Rees[◊], Øyvind Halskau[§,*]

[§]*Department of Molecular Biology, University of Bergen, Thormøhlensgate 55, NO-5006, Bergen, Norway.*

[†]*Centre for Biomolecular Sciences, University of Nottingham, University Park, Nottingham NG7 2RD, United Kingdom.*

[‡]*Department of Chemistry, University of Oslo, P. O. Box 1033, Blindern, NO-0315 Oslo, Norway.*

[◊]*School of Biosciences, University of Nottingham, Sutton Bonington Campus, Nottinghamshire, LE12 5RD, United Kingdom.*

**oyvind.halskau@uib.no. Tel. +47 55 58 45 63.*

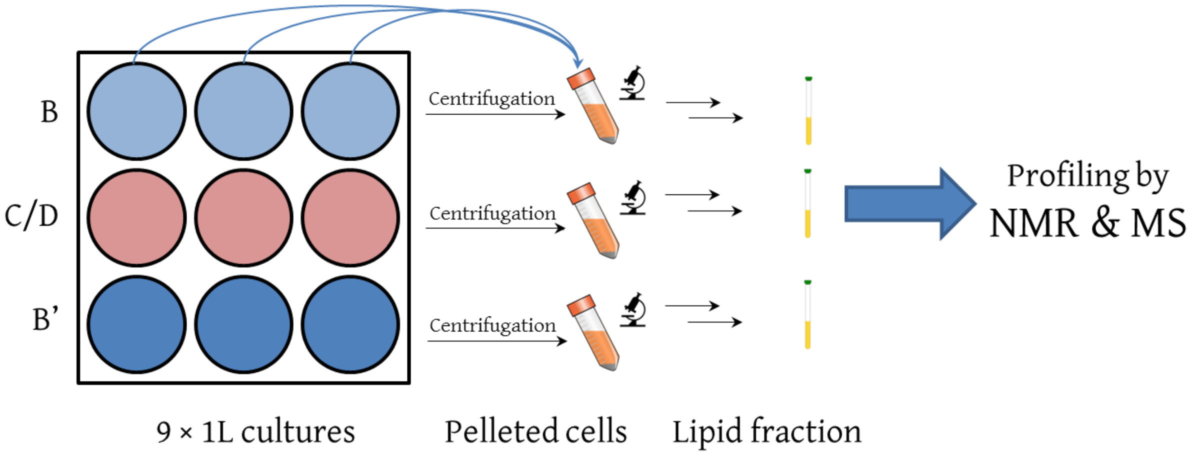

Figure S1. Experimental steps following preparation of the exponentially growing cultures. After harvesting, each culture was pelleted. Microscopy was performed to determine the morphology of the cells under study and for measurement of cell length and calculation of cell length distribution. Pelleting was followed by digestion of the cell wall in the presence of lipase inhibitors. The resulting material was then freeze-dried and the lipid fraction isolated. Finally, the lipid fraction was dispersed in CUBO solvent[15,35,49,76-78] for profiling using ^{31}P NMR and in isopropanol/dichloromethane (1:1 v/v) for profiling using MS.

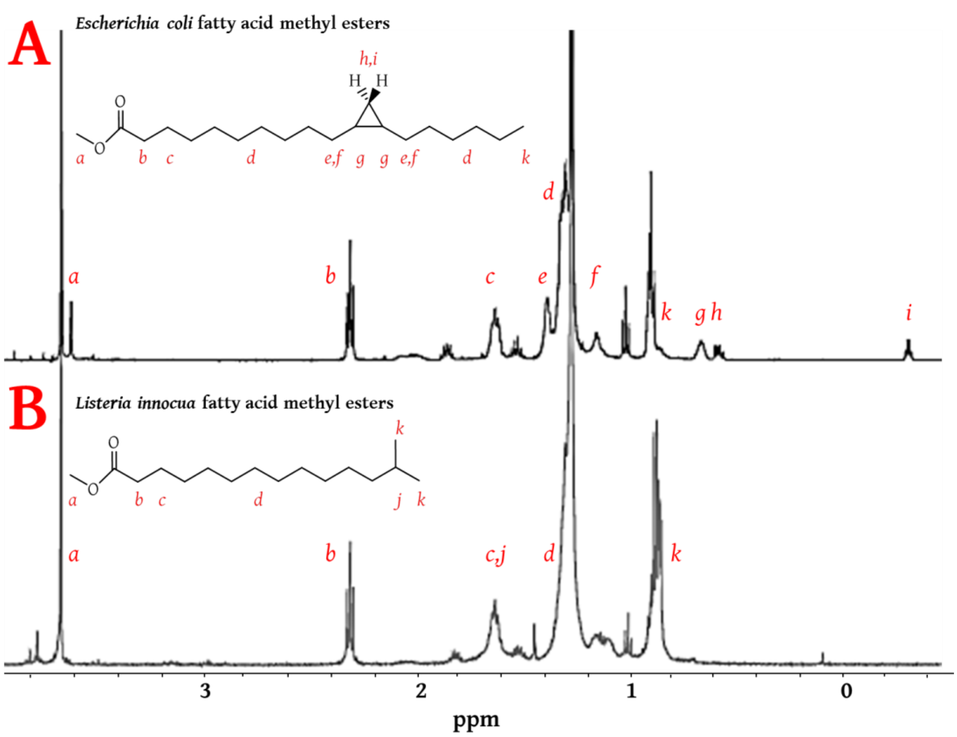

Figure S2. [1]*H NMR traces of fatty acid methyl esters isolated from lipid fractions of* E. coli *(trace A) and* L. innocua *(trace B), with example structures shown inset. The resonances corresponding to particular proton nuclei are marked (*a-k*), with a key on inset structures. The integrations of the appropriate signals indicate that around 40% of the FARs in the* E. coli *sample possess a cyclopropyl ring (*b *compared to* g,h,i*) and that the average number of methyl branches per FAR in* Listeria *is at least one (*b *compared to* k*).*

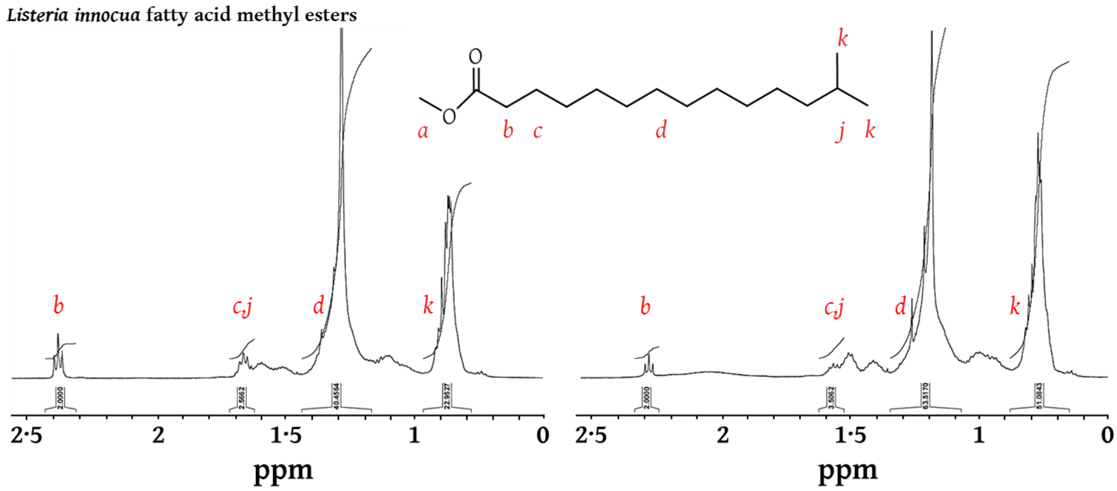

Figure S3. Two representative [1]*H NMR spectra of chloroform-soluble material from the dried and acid-treated remaining (aqueous/precipitate) fraction of the lipid isolation. The effectiveness of the isolation procedure developed in this study was tested by an attempt to isolate remaining fatty acid residues. The aqueous solution and precipitate were dried together and then treated with strong acid in order to hydrolyse any ester bond present, before being dried and washed with chloroform. The dried oil was subject to* [1]*H NMR spectroscopy. The integrations of the signals are calibrated by the α-CH_2 signal (signal* b*, 2.3-2.4 ppm). This indicates that the integration of other signals (*e.g. *methyl signals,* k*, 0.6-0.8 ppm) are much higher than can be ascribed to FAMEs alone (~25H. The integration of one methyl group is 3H, indicating that there was an average of 8 protons in this chemical environment per FAR present). The complexity of the methyl signal and others also suggests the presence of other (lipophilic) species. The fraction of FAs present (25H/8H) was therefore judged as ~30%* w/w *(mean* n = 4 *measurements).*

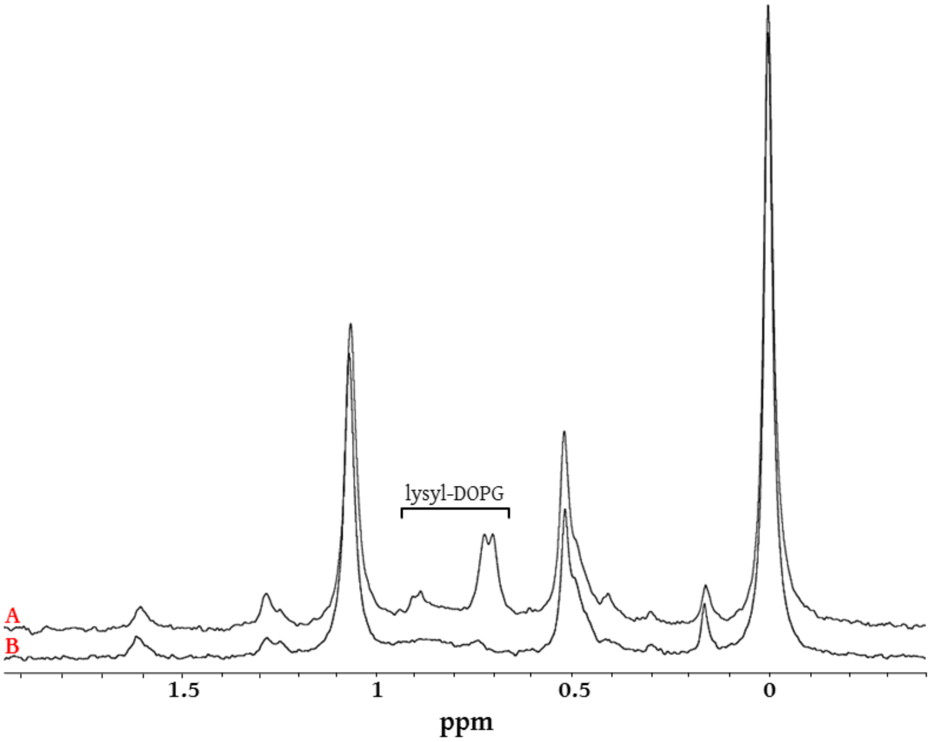

Figure S4. ^{31}P NMR (323.96 MHz) spectra for indicating lysyl-phosphatidylglycerol in lipid mixtures. Stacked traces of lecithin from Helianthus *mixed with lysyl-DOPG (A) and as supplied (B), indicating that the resonances for the two structural isomers of lysyl-DOPG are at ~0.7 and 0.9 ppm, representing the isomers with the lysyl groups on the primary and secondary hydroxyls of the head group's glyceryl moiety, respectively.*

	Cell cycle profile						*Phospholipid profiling*	
	B period ***(4h)***		*B′ period* *(6h)*		***C/D boundary***		^{31}*P NMR* *shift (ppm)*	*MS**
Cell length (µm, p = <0.001)	**1.24**	**± 0.29**	1.22	± 0.37	**1.92**	**± 0.44**	-	-
OD_{620} at harvest	**0.50**	**± 0.14**	1.57	± 0.18	**0.79**	**± 0.05**	-	-
**PG**	**32.7 %**	**± 8.4 %**	50.9 %	± 10.6 %	**43.3 %**	**± 14.4 %**	1.23-1.28	**637.4079** (C11:0/15:0); **665.4399** (C14:0/14:0); **679.4556** (C14:0/15:0); **693.4718** (C15:0/15:0); **705.4712** (C15:0/16:1); **707.4874** (C15:0/16:0); **721.5030** (C15:0/17:0; C16:0/16:0); **733.5025** (C15:0/18:1); **735.5187** (C16:0/17:0); **749.5338** (C17:0/17:0)
**CL**	**27.6 %**	**± 4.8 %**	20.1 %	± 5.1 %	**15.6 %**	**± 5.8 %**	0.77	**1098.7113** (C15:0/15:0/17:0/0:0); **1127.7504** (C17:0/15:0/17:0/0:0; C19:0/15:0/15:0/0:0). **1238.8314** (C13:0/15:0/13:0/15:0); **1294.8936** (C11:0/17:0/15:0/17:0; C13:0/18:0/14:0/15:0; C14:0/14:0/18:0/14:0; C15:0/15:0/15:0/15:0); **1308.9093** (C14:0/16:0/15:0/16:0; C15:0/15:0/16:0/15:0; C14:0/16:0/15:0/16:0); **1322.9252** (C14:0/16:0/17:0/15:0; C15:0/15:0/17:0/15:0; C14:0/16:0/16:0/16:0; C15:0/15:0/16:0/16:0); **1334.9249** (C16:1/15:0/17:0/15:0); **1336.9405** (14:0/17:0/16:0/16:0; 15:0/16:0/16:0/16:0; C15:0/16:0/17:0/15:0; C16:0/15:0/17:0/15:0); **1350.9564** (C14:0/18:0/17:0/15:0; C15:0/15:0/19:0/15:0; C15:0/15:0/17:0/17:0; C15:0/17:0/17:0/15:0; C16:0/16:0/16:0/16:0; C16:0/16:0/17:0/15:0; C17:0/15:0/17:0/15:0); **1362.9562** (C17:0/15:0/18:1/15:0); **1364.9719** (C16:0/16:0/17:0/16:0); **1378.9875** (C15:0/17:0/17:0/17:0);
**PE**	**19.6 %**	**± 6.2 %**	12.5 %	± 8.7 %	**7.8 %**	**± 4.3 %**	0.05, 0.10, 0.22, 0.31, 0.58[a]	**690.5084** (15:0/17:0); **704.5241** (C16:0/17:0); **718.5398** (C17:0/17:0; C15:/19:0)
lysyl-PG†	**2.8 %**	**± 3.1 %**	5.8 %	± 3.4 %	**4.3 %**	**± 4.1 %**	0.69	**807.5506** (C14:0/15:0); **821.5668** (15:0/15:0); **835.5824** (C15:0/16:0); **849.5980** (C15:0/17:0; C16:0/16:0); **877.6288** (C17:0/17:0)
lyso-PA	**0.6 %**	**± 0.7 %**	0.3 %	± 0.4 %	**1.8 %**	**± 1.0 %**	6.0-6.3[b]	**423.2510** (C17:0); 451.2823 (C19:0)
PA	**2.5 %**	**± 3.4 %**	3.5 %	± 2.0 %	**5.0 %**	**± 3.6 %**	4.8-5.3[b]	**647.4663** (C15:0/17:0); 661.4819 (C16:0/17:0); **675.4976** (C15:0/19:0; C17:0/17:0); **703.5283** (C17:0/19:0)
lysyl-CL‡	**4.4 %**	**± 2.9 %**	1.6 %	± 2.3 %	**13.1 %**	**± 12.7 %**	0.63‡	**1395.8457** (C58:0); **1409.8723** (C59:0); **1423.8990** (C60:0); **1437.9256** (C61:0)
PS	**0.7 %**	**± 1.4 %**	0.8 %	± 0.9 %	**0.4 %**	**± 0.3 %**	0.52	**706.0507** (C15:0/15:0); **721.4888** (C15:0/16:0; C14:0/17:0); **734.4937** (C15:0/17:0); **748.5093** (C16:0/17:0); **762.5250** (C17:0/17:0); **790.5604** (C17:0/19:0)
lyso-PG	**n/d**		3.5 %	± 3.2 %	**2.0 %**	**± 2.0 %**		**456.2488** (C14:0); **470.2644** (C15:0); **484.2801** (C16:0); **498.2957** (C17:0).
Others	**<5 %**		<5 %		**<5 %**		Var.	-

Table S1. Cell length and lipid head group profile of L. innocua *NCTC 11288. Cell length distributions determined through measurement of cells on micrographs (*Fig. *1, p = <0.01, independent samples students' t-test), lipid profile determined using ^{31}P NMR and MS from* n *= 5 isolates. *HRMS/MS performed in positive ion mode; masses given are the* m/z *less the adducts. Assignments in ^{31}P spectra were made using literature references[35,76-78] and †stacked 1D data from this study (Fig. S4). The FAs identified by MS are consistent with previous studies of* Listeria spp.[51,79,80]. *‡Tentative assignment. [a]PE exhibits several shifts* in vitro *due to interactions with the solvent and as a function of* pH *and concentration. [b]The shift of phosphate* mono-*ester-containing lipids is* pH *dependent.*

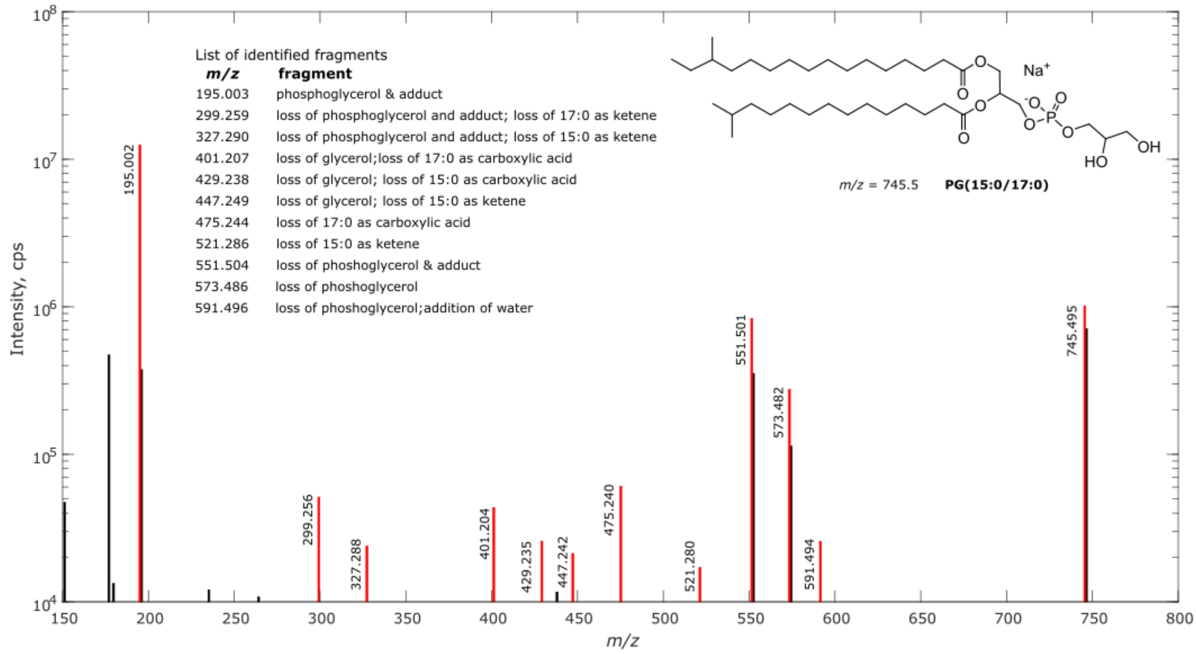

Figure S5. Representative fragmentation spectrum of a lipid (isoform PG(15:0, 17:0)) from Listeria innocua*. Only spectra in which fragmentation that described the lipid unambiguously were used to support identification. The data was acquired on an OrbiTrap Dionex 3000, (Waltham, MA, USA). Briefly, dry lipid mixtures were solubilized using a dichloromethane/isopropanol 1:1 mixture. The samples from this solution was injected (10 µL) and lipids separated by chromatography using a UPLC C18 column (1.7 µm particle size, Waters, 40°C, and flow rate of 0.4 mL/min). Ions were detected in positive mode, and MS data were analysed using software from Kochen* et al.[75], and Matlab code developed by authors. *Only spectra in which fragmentation that described the lipid unambiguously were used to support identification.*

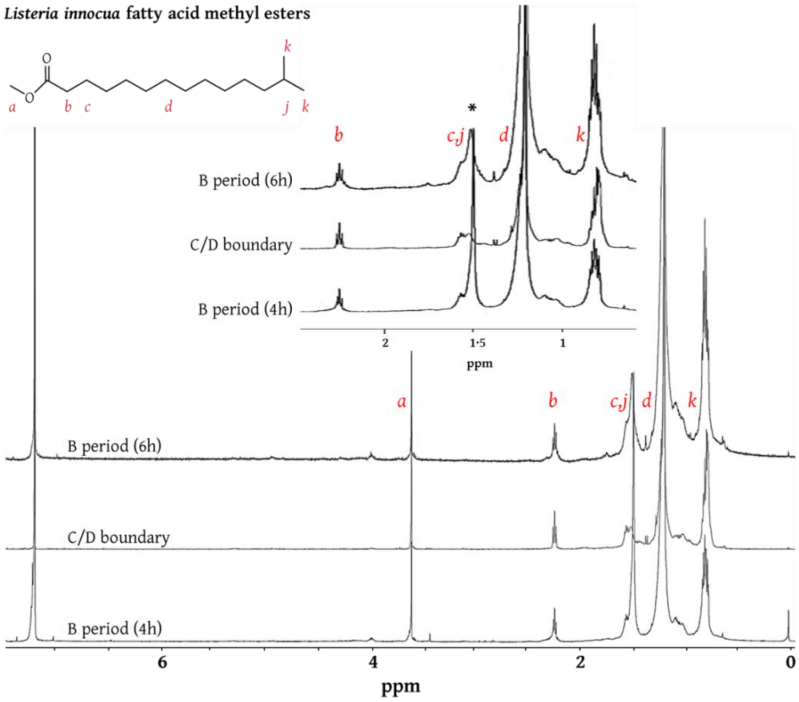

*Figure S6. Representative ^{1}H NMR spectra of fatty acid methyl ester preparations of lipid fractions isolated used for head group profiling. Resonances as described in the Experimental, with example labelled as inset structure. *resonance pertaining to grease (1.45 ppm), that overlaps with that of the β-CH_2 signal (c, 1.6 ppm). There was no evidence for the cyclopropyl groups typical of Gram-negative bacteria in any of the* Listeria *samples used.*

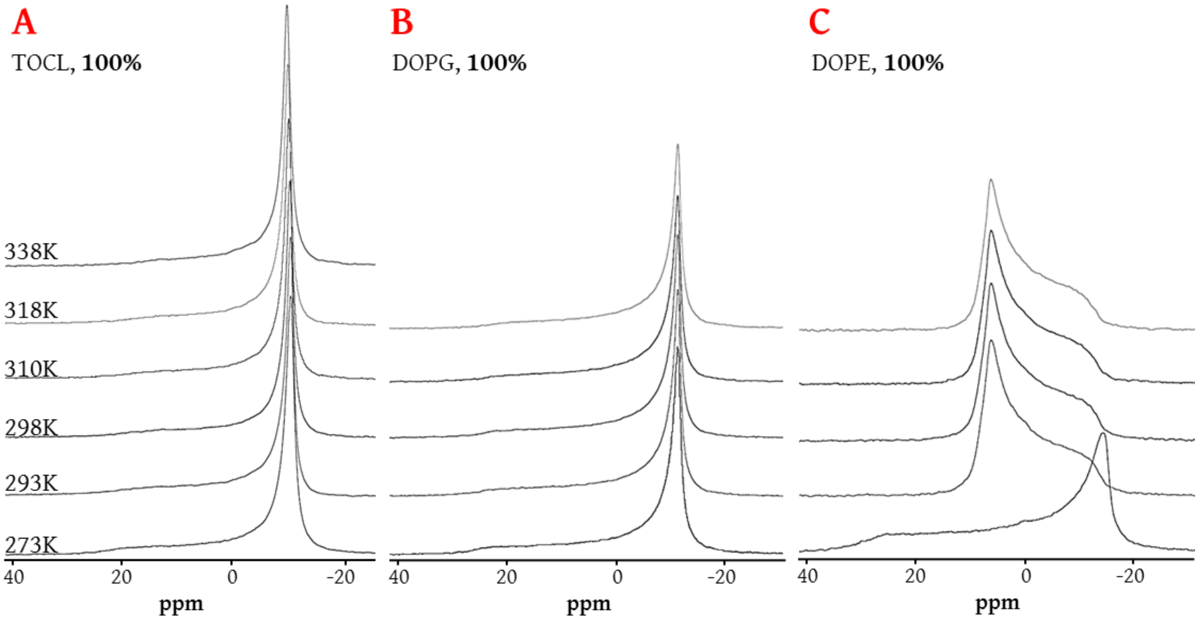

Figure S7. Broad line ^{31}P NMR temperature scans of hydrated single-lipid systems. A, TOCL; B, DOPG; C, DOPE.

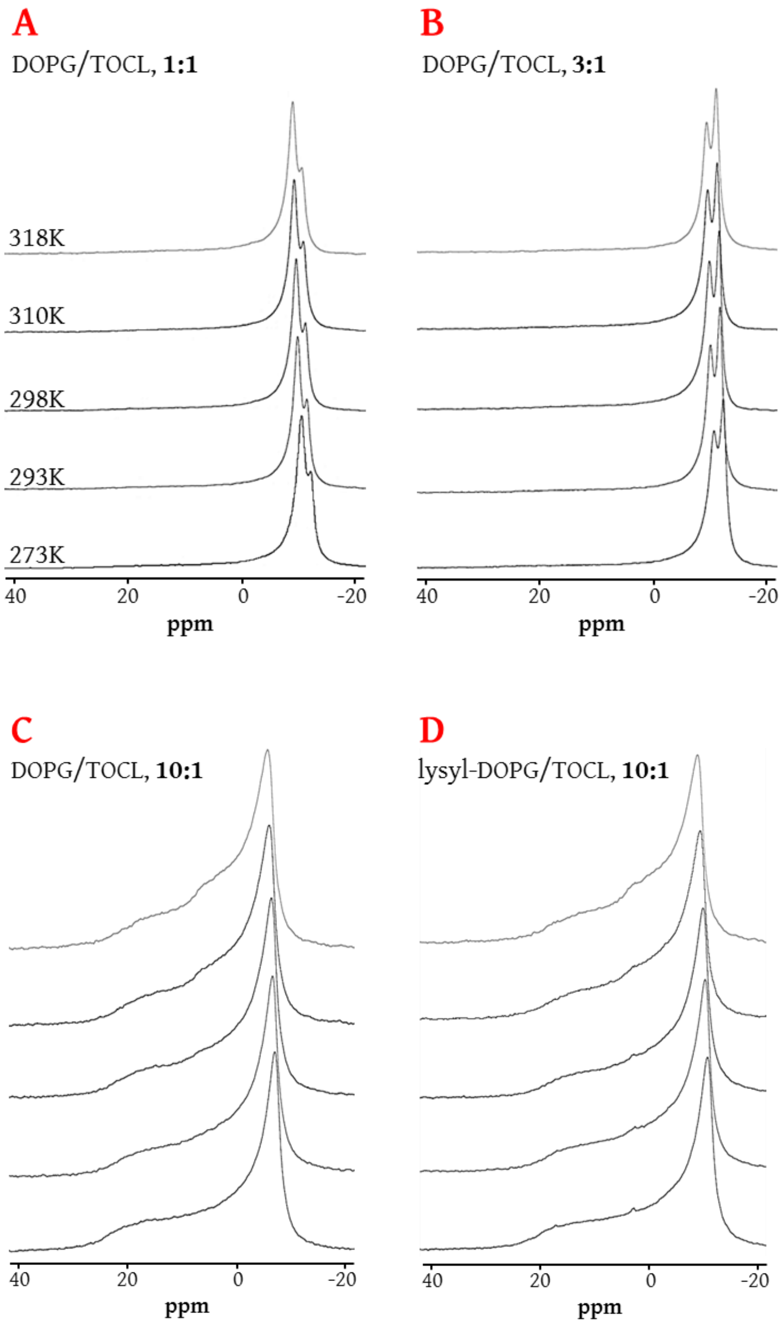

Figure S8. Broad line ^{31}P NMR (161.98 MHz) temperature scans of hydrated mixtures of DOPG and TOCL (A-C) and lysyl-DOPG and TOCL (D). A, 1:1 molar ratio of DOPG and TOCL; B, 3:1 molar ratio; C, 10:1 molar ratio; D, 10:1 molar ratio of TOCL and lysyl-DOPG.

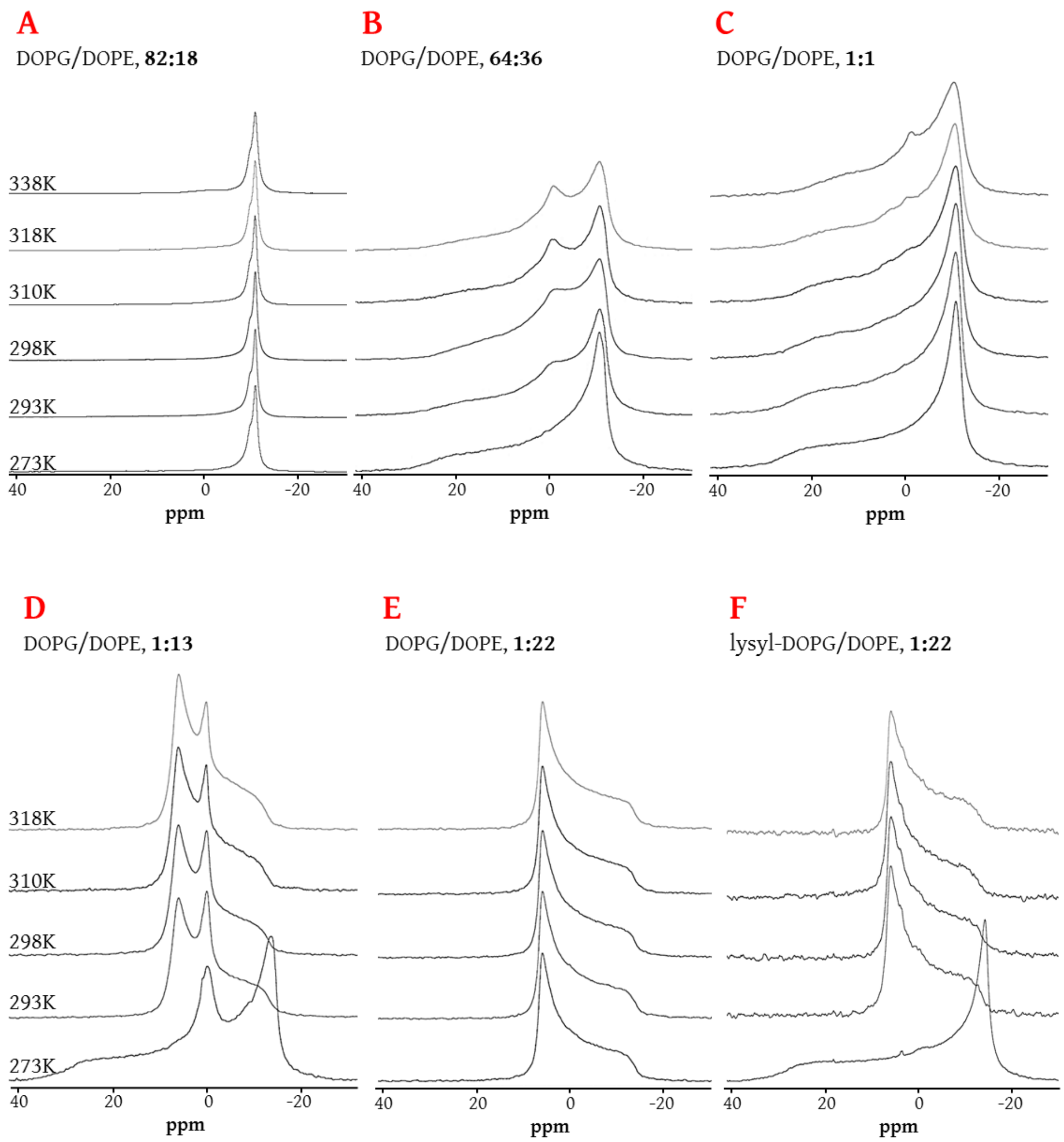

Figure S9. Broad line ^{31}P NMR (161.98 MHz) temperature scans of hydrated mixtures of DOPG and DOPE (A-E) and lysyl-DOPG and DOPE (F). A, 82:18 molar ratio of DOPG and DOPE; B, 36:64 molar ratio; C, 1:1 molar ratio; D, 1:13 molar ratio; E, 1:22 molar ratio; F, 1:22 molar ration of lysyl-DOPG and PE.

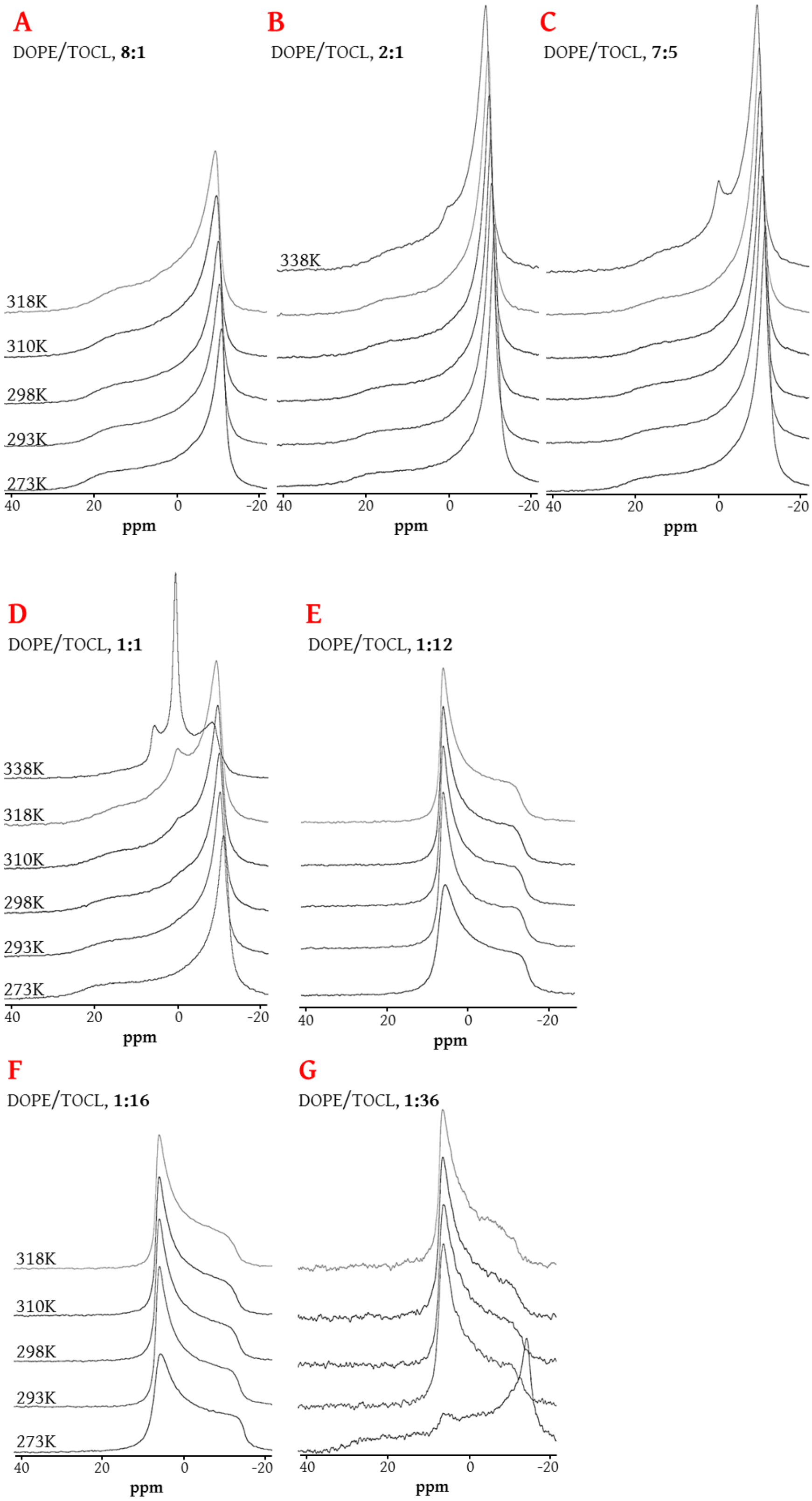

Figure S10. Broad line ^{31}P NMR (161.98 MHz) temperature scans of hydrated mixtures of TOCL and DOPE. A, 8:1 molar ratio of TOCL and DOPE; B, 2:1 molar ratio; C, 7:5 molar ratio; D, 1:1 molar ratio; E, 1:12; F, 1:16; G, 1:36.
